# Quackery in China

**Published:** 1895-09

**Authors:** 


### Quackery in China.

In the far-off province of Yun-nan of southwestern China there are divisions in medical practice not unlike those America and Europe know. There are "regulars" and there are quacks—and it is difficult to tell which of the two are the more unsafe. A missionary writes to one of our cotemporaries: "Quack doctors are commonly to be met with at very fair and on every market day, and they interfere not a little with the regular and resident practitioners of the city. So far as the people are concerned it makes but little difference which of them treats their cases for the local and the itinerant doctors are alike ignorant of the first principles of anatomy, physiology or medicine."

